# Traumatic arteriovenous fistula in the temporal region: a therapeutic challenge

**DOI:** 10.1590/1677-5449.200055

**Published:** 2021-08-02

**Authors:** Oona Tomiê Daronch, Pedro Henrique Bragato, Luiz Fernando Tosi Ferreira, Bárbara D’Agnoluzzo Moreira, Paulo Henrique Stahlke

**Affiliations:** 1 Universidade Federal do Paraná – UFPR, Hospital de Clínicas, Curitiba, PR, Brasil.

**Keywords:** arteriovenous fistula, trauma, temporal bone

## Abstract

Arteriovenous fistulas can be congenital or traumatic, the former being more common and diagnosed in childhood, and the latter being rarer and diagnosed later in life. Both require interventional treatment, which may be endovascular, or surgical repair and each case must be studied individually. This article presents the case of a 46-year-old patient with an arteriovenous fistula (AVF) between the left temporal artery and its corresponding veins resulting from a blunt trauma to the parietal region during childhood. The diagnosis was confirmed by imaging examination and he underwent conventional surgical treatment with improvement of symptoms. The case calls attention to a rare condition, its diagnostic investigation, and therapeutic approaches. The incidence of traumatic arteriovenous fistulas is low. They can occur in a variety of ways and can cause symptoms, requiring treatment, which is sometimes challenging, resulting in improvement in the patient's quality of life.

## INTRODUCTION

Arteriovenous fistulas (AVF) result from direct communication between an artery and a vein and have a number of different causes. They can emerge as malformations present from infancy, they may be created artificially to enable hemodialysis in chronic renal patients, or they can be traumatic.^1^ Traumatic vascular injuries are a surgical challenge. AVFs caused by vascular trauma are frequently the consequence of penetrating trauma and are more rarely caused by blunt traumas; they may be diagnosed many years after the traumatic event.^2^ The principal etiology is penetrating trauma, in 90% of cases, with blunt traumas responsible for the remaining 10% of cases. Percutaneous interventions, such as renal biopsies, cardiac catheterization, and orthopedic procedures are iatrogenic etiologies.^3^ The protocol for this study was approved by the Ethics Committee at our institution (CAAE: 38056920.0.0000.0096, decision number 4.340.540).

## PART I – CLINICAL SITUATION

The patient, J. L. K., a 46-year-old male with no comorbidities or previous surgery, was admitted for acute abdominal obstruction, probably due to congenital adhesion bands. He underwent explorative laparotomy with release of the adhesion bands, and recovered well during the postoperative period. While in hospital, he complained of headaches and described a history of blunt trauma to the left temporal area while playing rugby as a child, which had later progressed with recurrent episodes of headaches. He stated that the crises had gradually become more frequent, with no other relevant symptoms.

**Figure 1:**
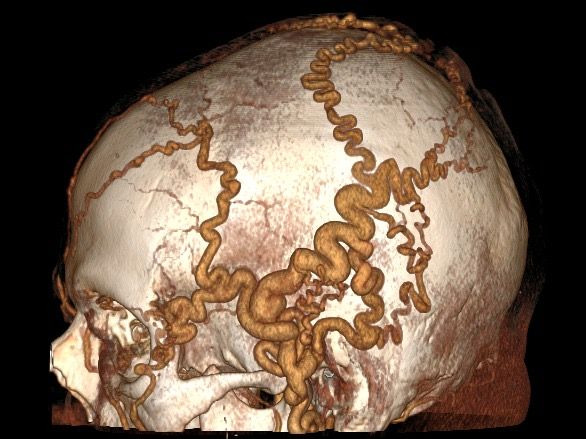
Preoperative lateral angiotomography showing arteriovenous fistula.

**Figure 2:**
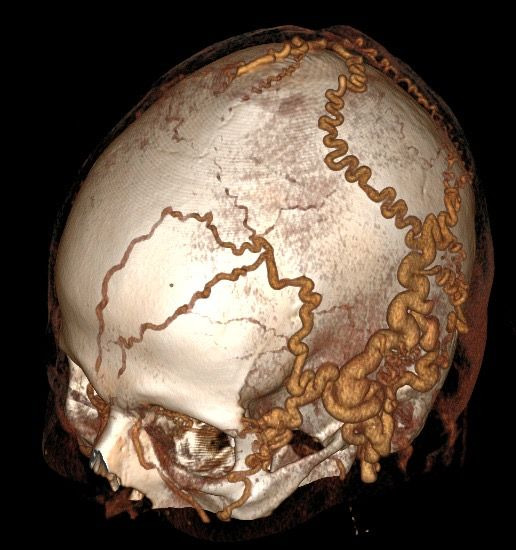
Preoperative anterolateral angiotomography showing arteriovenous fistula.

Physical examination found thrill on the patient’s scalp, from the left temporal region to the left occipital region, and dilatation of the venous system in the same territory. The AVF had no cardiac repercussions, according to the echocardiogram, probably because of its small size. Investigation proceeded with angiotomography, which demonstrated accentuated dilatation and tortuosity of superficial arterial and venous vessels of the head, primarily on the left, and signs suggestive of an extensive AVF in the left temporoparietal area, with no communication with deep or intracranial vessels (Figure 1 and 2).

## PART II – WHAT WAS DONE

The decision was taken to conduct surgical treatment to close the AVF while still in hospital, since this option is freely available and, in this case, embolization would have needed a large quantity of embolization agent. Ligature of the left superficial temporal artery was performed proximal to the fistula. Immediately after ligature, the thrill completely disappeared and venous system engorgement reduced considerably. The AVF was identified prior to ligation by inspection and palpation. The imaging exams employed enabled diagnostic confirmation and definition of the extent of the AVF.

**Figure 3:**
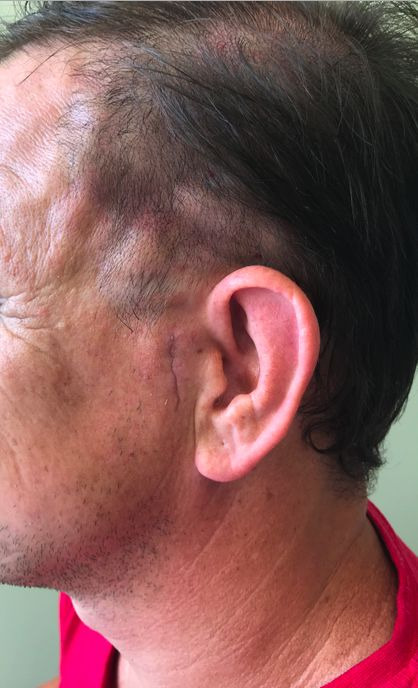
Surgical incision site, showing appearance after healing.

Since facial vascularization involves countless anastomoses and considerable collateral circulation, there was no impairment of tissue perfusion. With no complications related to either of the procedures, the patient was discharged during the immediate postoperative period after closure of the AVF, five days after the explorative laparotomy and release of adhesion bands. At 7-day and 30-day outpatients follow-up consultations, the patient reported considerable improvement in the headache episodes and his clinical course after discharge was good. The surgical access site and postoperative healing are shown in Figure 3.

## DISCUSSION

An AVF was first described in the literature in 1757,^3^ and the first attempt at surgical repair was described by Breschet in 1837, employing arterial ligature proximal of the fistula. This technique remains in use today, as illustrated by the surgery performed in the case reported here. Arteriovenous fistulas occur when the artery wall or its vasa vasorum are ruptured, with the result that arterial endothelium proliferates into the adjacent veins. As such, AVF can occur either because of abnormal repair of vessels or because of proliferation of endothelial sprouts from the vasa vasorum of vessels injured by traumatism. This mechanism may be responsible for some of the rare cases of spontaneous AVF of superficial temporal artery, but it is improbable that this is responsible for posttraumatic cases, in which symptoms appear immediately.^4^

Analyzing just those traumatic AVF that involve the scalp, it is found that the superficial temporal artery is the most often affected, along with the occipital artery and its corresponding veins.^2^ Whereas the intracranial vessels are most frequently affected in congenital cases, the opposite is true in traumatic cases, in which facial circulation is most affected because of its greater susceptibility, as described in the case reported here in which the patient had an AVF in the left temporal region.

Although trauma is a rare cause of AVF according to data in the literature,^4^ one case has been reported that was similar to the case in the present study, describing a male 23-year-old patient with a tortuous and pulsating mass in the left temporofrontal area 10 years after a blunt trauma to that region. Computed tomography with intravenous contrast and ultrasonography revealed a vascular mass with an AVF in soft tissues of this area only. Surgery was performed to completely excise the vascular mass; similar to the patient in this study, in whom the superficial temporal artery was ligated.

Another similar case report in the literature,^5^ with superficial temporal artery involvement due to trauma was a 24-year-old patient who developed an epidural hematoma on the right surface of the frontal lobe after a traffic accident and was treated urgently with surgical resection.

Occurrence of symptoms is linked to the pathophysiologic mechanism of AVF formation and the process can occur over the course of 1 year, depending on the complexity of the case.^3^ An anomalous arteriovenous communication triggers considerable clinical manifestations. In the case of an AVF in the scalp, the most common symptoms are localized headaches, pulsating mass, and intense buzzing. The reduced circulation to tissues can also result in skin changes, including hair loss and necrosis.

Symptoms generally manifest early in cases of local trauma, as described by Miekisiak et al.,^6^ who reported the case of a 30-year-old male patient who had a visibly pulsating mass and complained of intense buzzing in the left ear after a car accident. He underwent arteriography for diagnosis and opted for surgical treatment, taking into account the high cost of endovascular management of such large injuries combined with the significant risk of only achieving partial embolization, similar to the treatment performed in the case described in this article.

It is known that pulses distal of the AVF may be present, but, in fistula cases with significant arteriovenous shunts, the arterial blood is diverted away from extremities and signs of distal ischemia may be present, especially in the limbs.^7^ The Nicoladoni-Israel-Branham sign may also be present, which is bradycardia provoked by manual compression of the artery proximal to the AVF, although this sign was negative in the case described here.

Arteriography is the gold standard for diagnosis, but angiotomography and Doppler ultrasound also have an important role to play.^7^ Arteriography is a dynamic study and will very often show the artery feeding the fistula at the exact point at which it communicates with the vein. The major disadvantages are the cost and complications inherent to invasive arterial access. Angiotomography is convenient because it is noninvasive, more accessible than arteriography, and offers similar results for diagnosis of an AVF.^8^ An additional factor is that angiotomography enables assessment of adjacent structures, which is important for planning surgery.

There are some possible differential diagnoses for traumatic AVF, such as arterial occlusion, dissection, and formation of pseudoaneurysms or true aneurysms.^8^ Arteriovenous fistulas of the superficial temporal artery have an estimated incidence^9^ of 0.5 to 2.0% of cases; while incidence of traumatic pseudoaneurysms varies^10^ from 4 to 13%. In the present case, although the trauma was blunt and the incidence of pseudoaneurysms exceeds that of AVF, angiotomography confirmed the diagnosis of AVF.

With regard to treatment, a period of 2 weeks is needed to indicate interventional treatment,^11^ since if the AVF does not undergo spontaneous regression, it is necessary to perform direct primary repair or anatomic reconstruction (repair with venous grafts). The main treatment options include endovascular treatment, which is minimally invasive, reduces hospital costs, and lessens lost work productivity.^11^ However, open surgery is the only option in hemodynamically unstable patients, those with contraindications for endovascular treatment, or in those for whom endovascular treatment has been unsuccessful.^3^

With regard to the treatment options for the present case, angioembolization of the lesion or surgery were discussed, but both involve risk of venous thrombosis of the veins that receive the arterial flow and, additionally, the location of the lesion in the temporal region could have made endovascular treatment difficult because of the anatomy and extensive vascularization. The principal postoperative complications are venous thrombosis of the veins that receive arterial flow.^12^ Postoperative follow-up of patients is not complex in general and the majority of reports result in good outcomes.^13^ In cases in which there is doubt about complete AVF closure, because of persistence of symptoms or direct signs on physical examination, these can be easily cleared up with Doppler or other invasive examinations. Failed endovascular treatment can be resolved with surgery, but further attempts using the same method initially employed are not ruled out.

Traumatic AVF is an uncommon vascular disorder. Although AVFs do not always manifest visible signs, they may be found during physical examination and should be investigated to plan treatment. Surgery was the option chosen for the patient in question and he exhibited clinical improvement of the symptomology presented before repair of the AVF, showing the safety and efficacy of this treatment method.
