# Supplementary material for: Traumatic arteriovenous fistula in the temporal region: a therapeutic challenge
Source: J Vasc Bras. 2021 Aug 2;20:e20200055. doi: 10.1590/1677-5449.200055 (PMC8336982; doi:10.1590/1677-5449.200055)
Supplement: Translation

# Translation: Fístula arteriovenosa traumática em região temporal: um desafio terapêutico

**Authors:** Oona Tomiê Daronch, Pedro Henrique Bragato, Luiz Fernando Tosi Ferreira, Bárbara D’Agnoluzzo Moreira, Paulo Henrique Stahlke

**Affiliations:** 1 Universidade Federal do Paraná – UFPR, Hospital de Clínicas, Curitiba, PR, Brasil.

**Keywords:** fístula arteriovenosa, trauma, osso temporal

## Abstract

As fistulas arteriovenosas podem ser congênitas ou traumáticas, sendo as primeiras mais comuns e diagnosticadas na infância e as últimas mais raras e com diagnóstico mais tardio. Ambas necessitam de tratamento intervencionista, que pode ser endovascular ou correção cirúrgica, sendo que cada caso deve ser estudado individualmente. Este artigo apresenta o caso de um paciente de 46 anos, com fístula arteriovenosa na artéria temporal superficial esquerda com suas veias correspondentes decorrente de trauma contuso na região temporal na infância. O diagnóstico foi confirmado por exame de imagem, e o paciente foi submetido a tratamento cirúrgico convencional, apresentando melhora dos sintomas. O caso chama atenção para uma afecção rara, sua investigação diagnóstica e condutas terapêuticas. As fistulas arteriovenosas traumáticas apresentam baixa incidência, ocorrem em variadas localizações e podem causar sintomas, requerendo tratamento, às vezes desafiador, com melhora da qualidade de vida do paciente.

## INTRODUÇÃO

As fístulas arteriovenosas (FAV) resultam de uma comunicação direta entre uma artéria e uma veia e apresentam diferentes origens, podendo surgir como malformações desde a infância, ser artificialmente confeccionadas para possibilitar a hemodiálise em doentes renais crônicos ou ainda ser traumáticas^1^. As lesões vasculares traumáticas representam um desafio cirúrgico. As FAV decorrentes do trauma vascular frequentemente são consequência do trauma penetrante e mais raramente do trauma contuso, podendo ser diagnosticadas muitos anos depois do evento traumático^2^. A principal etiologia é o trauma penetrante em 90% dos casos, e o trauma contuso é responsável pelos outros 10% dos casos. As intervenções percutâneas, como biópsias renais, cateterismo cardíaco e procedimentos ortopédicos, representam a etiologia iatrogênica^3^. O protocolo foi aprovado pelo Comitê de Ética de nossa instituição (CAAE: 38056920.0.0000.0096, parecer nº 4.340.540).

## PARTE I – SITUAÇÃO CLÍNICA

Paciente J. L. K., masculino, de 46 anos, sem comorbidades ou cirurgias prévias, foi internado com quadro de abdome agudo obstrutivo, provavelmente por bridas congênitas. Foi submetido a laparotomia exploradora com lise de bridas, apresentando boa evolução no pós-operatório. Durante o internamento, queixou-se de cefaleia e referiu história de trauma contuso em região temporal esquerda durante jogo de rúgbi na infância, o qual evoluiu posteriormente com episódios recorrentes de cefaleia. Relatou que as crises foram tornando-se gradativamente mais frequentes, sem outros sintomas relevantes associados.

Ao exame físico, identificou-se a presença de frêmito no couro cabeludo, desde a região temporal esquerda até a região occipital esquerda, bem como dilatação do sistema venoso na mesma topografia. A FAV não apresentava repercussão cardíaca segundo o ecocardiograma, provavelmente por seu pequeno tamanho. Foi, então, realizada investigação com angiotomografia, que demonstrou presença de acentuada dilatação e tortuosidade de ramos arteriais e venosos superficiais do crânio, principalmente à esquerda, e sinais sugestivos de extensa FAV na região temporoparietal esquerda sem comunicação com ramos profundos ou intracranianos (Figura 1 e 2).

**Figura 1:** Angiotomografia pré-operatória lateral demonstrando fístula arteriovenosa.

**Figura 2:** Angiotomografia pré-operatória anterolateral demonstrando fístula arteriovenosa.

## PARTE II – O QUE FOI FEITO

Optou-se pela realização do tratamento cirúrgico para fechamento da FAV no mesmo internamento, pois essa opção é amplamente disponível, e a embolização nesse caso demandaria grande quantidade de agente embolizante. Realizou-se ligadura da artéria temporal superficial esquerda proximal à fístula. Imediatamente após a ligadura, houve desaparecimento completo do frêmito e redução considerável do ingurgitamento do sistema venoso. Para identificação e ligadura da FAV, foram realizadas inspeção e palpação. Os exames de imagem empregados possibilitaram a confirmação diagnóstica e definiram a extensão da FAV.

Tendo em vista que a vascularização da face apresenta inúmeras anastomoses e circulação colateral, não houve prejuízo à perfusão tecidual. Sem complicações advindas de ambos os procedimentos realizados, o paciente recebeu alta no pós-operatório imediato do fechamento da FAV e no quinto dia de pós-operatório da laparotomia exploradora com lise de bridas. Nos retornos ambulatoriais no sétimo e trigésimo dias de pós-operatório, referiu melhora importante dos episódios de cefaleia e apresentou boa evolução após a alta. O local de acesso cirúrgico e a cicatriz pós-operatória podem ser visualizados na Figura 3.

**Figura 3:** Local onde foi realizada incisão cirúrgica e aspecto da cicatriz.

## DISCUSSÃO

A primeira descrição^3^ de FAV na literatura foi feita em 1757, e a primeira tentativa de correção cirúrgica foi descrita por Breschet em 1837, que procedeu à ligadura arterial proximalmente à fistula. Essa técnica apresenta utilidade até os dias atuais, tendo em vista a cirurgia realizada no presente caso em estudo. As FAV ocorrem quando a parede arterial ou seus *vasa vasorum* são rompidos, levando o endotélio arterial a proliferar nas veias adjacentes. Dessa forma, as FAV podem ocorrer tanto por conta da reparação anormal dos vasos quanto por uma proliferação de brotos endoteliais a partir da *vasa vasorum* dos vasos lesados pelo traumatismo. Esse mecanismo pode ser responsável por alguns casos raros de FAV da artéria temporal superficial espontânea, mas é improvável que seja responsável por casos pós-traumáticos, em que o aparecimento dos sintomas é imediato^4^.

Ao analisar as FAV traumáticas apenas do couro cabeludo, percebeu-se que a artéria temporal superficial é a mais envolvida, bem como a artéria occipital e suas veias correspondentes^2^. Apesar de nos casos congênitos, os vasos intracranianos serem mais acometidos, em casos de trauma ocorre o contrário, sendo a circulação da face mais afetada, por apresentar maior suscetibilidade, como relatado nesse caso, uma vez que o paciente apresentou FAV na região temporal esquerda.

Apesar de trauma ser uma causa rara de FAV segundo dados da literatura^4^, foi relatado um caso similar ao do presente estudo, evidenciando um paciente masculino de 23 anos com massa tortuosa e pulsátil na área temporofrontal esquerda 10 anos após trauma contuso naquela área. A tomografia computadorizada com contraste intravenoso e a ultrassonografia revelaram massa vascular com FAV em tecidos moles somente nessa área. Foi realizada cirurgia com excisão completa da massa vascular, de forma semelhante ao paciente em estudo, no qual foi realizada ligadura da artéria temporal superficial.

Outro relato de caso semelhante^5^ no caso de envolvimento de artéria temporal superficial por trauma na literatura é de um paciente de 24 anos que, após acidente de trânsito, evoluiu com um hematoma epidural na superfície direita do lobo frontal, tratado urgentemente por ressecção cirúrgica.

A ocorrência de sintomas está relacionada com o mecanismo fisiopatológico da formação das FAV, e esse processo pode ocorrer durante até 1 ano dependendo da complexidade do caso^3^. Uma conexão arteriovenosa anômala resulta em uma rica manifestação clínica, sendo que os sintomas mais comuns de uma FAV no couro cabeludo são dor de cabeça localizada, massa pulsátil e intenso zumbido. Devido à diminuição da circulação dentro do tecido, também podem ocorrer alterações na pele, como calvície ou necrose.

Os sintomas geralmente tornam-se evidentes de forma mais precoce nos casos de trauma local, conforme descrito por Miekisiak et al.^6^, que relatam o caso de um paciente masculino, 30 anos, que apresentou massa visivelmente pulsátil e queixa de intenso zumbido na orelha esquerda após acidente automobilístico. Foi submetido a arteriografia para diagnóstico, optando-se por tratamento cirúrgico, levando em consideração o alto custo do manejo endovascular de lesões tão grandes, combinado com um risco significativo de embolização apenas parcial, similar ao tratamento realizado no presente artigo.

Sabe-se que pulsos distais à FAV podem estar presentes, mas, nos casos de fístulas com *shunt* arteriovenoso importante, há desvio de sangue arterial das extremidades e sinais de isquemia distal, especialmente nos membros^7^. O sinal de Nicoladoni-Israel-Branham também pode estar presente, que é a bradicardia causada pela compressão manual da artéria proximal à FAV, porém ele não estava positivo no caso deste trabalho.

No diagnóstico, a arteriografia é o padrão-ouro, mas a angiotomografia e o Doppler também desempenham um papel de destaque^7^. A arteriografia é um estudo dinâmico e evidencia muitas vezes a artéria alimentando a fístula no seu exato ponto de comunicação com a veia. As principais desvantagens são os custos e as complicações inerentes ao acesso arterial invasivo. A angiotomografia é conveniente por não ser invasiva, ser mais acessível que a arteriografia e fornecer resultados semelhantes para o diagnóstico de FAV^8^. Um fator adicional é que a angiotomografia permite a avaliação de estruturas adjacentes, importante para o planejamento cirúrgico.

Existem possíveis diagnósticos diferenciais para a FAV traumática, como oclusão arterial, dissecção e formação de pseudoaneurisma ou aneurismas verdadeiros^8^. As FAV da artéria temporal superficial apresentam incidência estimada^9^ entre 0,5 a 2,0% dos casos; já a incidência de pseudoaneurisma traumático varia^10^ de 4 a 13%. No presente caso, apesar de o trauma ser contuso e de a incidência de pseudoaneurismas ser superior à FAV, a angiotomografia confirmou o diagnóstico.

Sobre o tratamento, o período necessário para indicar tratamento intervencionista é de 2 semanas^11^, pois, se a FAV não apresentar regressão espontânea, há necessidade de realizar reparo primário direto ou reconstrução anatômica (reparo com enxerto venoso). As principais opções terapêuticas são a abordagem endovascular, com natureza minimamente invasiva, redução de custos hospitalares e diminuição da perda de produtividade no trabalho^11^. Em contrapartida, a cirurgia aberta torna-se a única opção em pacientes hemodinamicamente instáveis, contraindicados para tratamento endovascular ou nos quais o tratamento endovascular não teve êxito^3^.

Em relação às possibilidades terapêuticas para o presente caso, foram discutidas a angioembolização da lesão ou a realização de cirurgia, porém em ambas havia o risco de trombose venosa das veias que recebiam o fluxo arterial, e, além disso, a localização da lesão na região temporal poderia dificultar o tratamento endovascular pela anatomia e ampla vascularização. As principais complicações pós-operatórias são trombose venosa das veias que recebiam o fluxo arterial^12^. O seguimento pós-operatório dos pacientes em geral não é complexo, sendo que a maioria dos relatos resultaram em bons desfechos^13^. Os casos de dúvida quanto ao fechamento completo da FAV, por manutenção dos sintomas ou por sinais diretos do exame físico, podem ser facilmente esclarecidos com o uso do Doppler ou outros exames invasivos. As falhas do tratamento endovascular podem ser resolvidas com abordagem cirúrgica, mas não impedem novas tentativas através da mesma modalidade inicialmente empregada.

A FAV traumática é uma alteração vascular pouco comum. Apesar de nem sempre manifestar sinais visíveis, pode ser um achado no exame físico e deve ser investigada para programação terapêutica. A cirurgia foi a opção de escolha para o paciente em questão, e ele apresentou melhora clínica da sintomatologia apresentada antes da correção da FAV, ressaltando a segurança e resolutividade dessa ferramenta terapêutica.
